# Ultrasound-guided erector spinae plane block versus serratus anterior plane block for analgesia and respiratory function in patients with multiple rib fractures: a large-sample, single-center, randomized, double-blind, controlled trial

**DOI:** 10.3389/fmed.2026.1826279

**Published:** 2026-06-05

**Authors:** Zhiliang Tang, Lina Zhou

**Affiliations:** 1 Department of Emergency, Shaoxing People’s Hospital, Shaoxing, Zhejiang, China; 2 Department of Anesthesiology, Shaoxing People’s Hospital, Shaoxing, Zhejiang, China

**Keywords:** analgesia, erector spinae plane block, randomized controlled trial, regional anesthesia, rib fractures, serratus anterior plane block

## Abstract

**Objective:**

This large-sample, randomized, double-blind controlled trial directly compared the analgesic efficacy and impact on respiratory function of ultrasound-guided erector spinae plane block (ESPB) versus serratus anterior plane block (SAPB) in patients with unilateral multiple rib fractures.

**Methods:**

A total of 158 eligible patients were randomized to receive either ESPB (*n* = 73) or SAPB (*n* = 85), performed with 0.375% ropivacaine. The primary outcome was the time-weighted area under the curve (AUC) of resting pain Numerical Rating Scale (NRS) scores over 24 h post-intervention. Secondary outcomes included dynamic pain scores, opioid consumption, respiratory function (diaphragmatic excursion, FVC, FEV1), recovery quality (QoR-15), and clinical outcomes.

**Results:**

The ESPB group demonstrated a significantly lower 24-h pain AUC compared to the SAPB group (mean difference −17.78, 95% CI: −21.00 to −14.56, *p* < 0.001), indicating superior comprehensive analgesia. ESPB also significantly reduced 24-h opioid consumption and improved QoR-15 scores (both *p* < 0.01). While both groups showed significant within-group improvement in respiratory function parameters, no significant between-group differences were found. Subgroup analysis revealed that the analgesic superiority of ESPB was consistent across different fracture locations but was more pronounced in patients with ≤5 fractures (interaction *p* = 0.023). Safety profiles and complication rates were comparable.

**Conclusion:**

For patients with unilateral multiple rib fractures, ESPB provides significantly better early comprehensive analgesia and reduces opioid requirements compared to SAPB, without compromising safety. ESPB may be considered as a preferred first-line regional analgesic technique, especially for patients with a lower fracture burden.

**Clinical trial registration:**

Chinese Clinical Trial Registry (ChiCTR), Registration No. ChiCTR2300067544.

## 1 Introduction

Blunt chest trauma represents a common critical condition in emergency and trauma centers. Among these injuries, multiple rib fractures (typically defined as fractures of three or more ribs) present a major focus and challenge in clinical management due to the severe pain and significant respiratory compromise they cause. Statistics indicate that rib fractures constitute a very high proportion of all blunt thoracic injuries (1). Furthermore, as the number of fractures increases, the patient’s risk of complications such as pneumonia and respiratory failure, along with mortality rates, shows a marked upward trend (2). This not only prolongs hospital stays but also escalates the economic burden of care. Consequently, for multiple rib fractures, the core of treatment transcends mere bony union and lies in implementing safe, potent, and sustained analgesia to disrupt the vicious cycle of “pain-hypoventilation-pulmonary complications.”

Traditional analgesic strategies have primarily relied on systemic opioid administration. However, the associated risks of respiratory depression, sedation, nausea and vomiting, and potential for dependency, particularly in elderly patients or those with comorbidities, may exacerbate the clinical situation (3). Regional analgesic techniques offer a superior alternative. Thoracic epidural analgesia was once considered the “gold standard,” but its high technical demands, risks of hypotension and nerve injury, and limitations in anticoagulated patients have restricted its widespread adoption in emergency departments and general wards (4). In recent years, ultrasound-guided fascial plane block techniques have rapidly advanced, revolutionizing pain management for rib fractures due to their relative simplicity of performance, high safety profile under visualization, and minimal physiological interference (5).

Among the various fascial plane blocks, the erector spinae plane block (ESPB) and the serratus anterior plane block (SAPB) are particularly prominent and have become hotspots in clinical research. The ESPB involves injecting local anesthetic between the deep fascia of the erector spinae muscle and the thoracic transverse processes (6). The solution spreads widely along the fascial plane, theoretically blocking both the dorsal and ventral rami of the spinal nerves, thereby providing extensive analgesic coverage for the posterior and posterolateral chest wall. The SAPB, in contrast, involves injecting the solution superficial or deep to the serratus anterior muscle, primarily targeting the lateral cutaneous branches of the intercostal nerves that innervate the anterolateral chest wall (7). Both techniques can be safely performed at the bedside or in the emergency room.

Although a considerable number of retrospective studies and some randomized controlled trials have individually confirmed the effectiveness of both ESPB (8) and SAPB (9) for rib fracture analgesia, a critical gap persists in the current evidence base: the lack of a direct, head-to-head comprehensive comparison of these two mainstream techniques within a unified, rigorous prospective study design (10). Existing research is often limited by small sample sizes or a narrow focus on single pain score outcomes (11). High-quality direct comparative evidence remains scarce regarding which technique yields superior benefits for patients with specific fracture patterns, especially concerning differences in their impact on key clinical hard endpoints such as respiratory function recovery and complication prevention (12). For instance, while some perspectives suggest ESPB may be more effective for posterior fractures and SAPB for anterolateral pain, this anatomy-based hypothesis urgently requires validation through high-level clinical research (13).

In light of this, the present study was designed and conducted as a single-center, large-sample, prospective, randomized, double-blind controlled trial (14). We aimed to move beyond simple pain score comparisons and systematically evaluate the effects of ESPB versus SAPB in patients with unilateral multiple rib fractures. Core comparative dimensions included: comprehensive analgesic efficacy within 24 h, the degree of improvement in diaphragmatic function and pulmonary ventilation, the opioid-sparing effect, patient-reported recovery quality, and the incidence of hospitalization-related complications (15). Based on anatomical principles, we hypothesized that ESPB might provide superior overall analgesic efficacy. Simultaneously, we planned to thoroughly investigate whether fracture characteristics (such as dominant location and number) might influence the differential effectiveness of the two techniques. The findings of this study will provide robust and nuanced evidence-based guidance for clinicians in selecting the most appropriate block technique for analgesia in multiple rib fractures, thereby aiming to optimize patient outcomes.

## 2 Materials and methods

### 2.1 Study design

This study was a single-center, prospective, randomized, double-blind, parallel-group, superiority clinical trial. The study protocol was developed in strict accordance with the ethical principles of the Declaration of Helsinki and received formal approval from the Ethics Committee prior to initiation. All participating patients were fully informed of the study’s purpose, procedures, potential risks, and benefits before enrollment and provided written informed consent. This trial was registered with the Chinese Clinical Trial Registry. The reporting of this study will adhere to the Consolidated Standards of Reporting Trials (CONSORT) statement. The registration number is ChiCTR2300067544.

### 2.2 Study participants

Participants were consecutive patients presenting with acute blunt chest trauma to the Emergency Department of our hospital between December 1, 2023, and December 31, 2025, who were subsequently admitted to the Trauma Center or Thoracic Surgery Ward. Patient inclusion and exclusion were rigorously screened by uniformly trained research coordinators based on the following criteria.

Inclusion criteria comprised: (1) Age ≥18 years; (2) diagnosis of unilateral, acute (time from injury to admission <48 h) multiple rib fractures (≥3 ribs) confirmed by 64-slice or higher multidetector computed tomography; (3) a resting-state Numerical Rating Scale (NRS) pain score ≥4 at admission without the use of potent analgesics; (4) American Society of Anesthesiologists (ASA) physical status classification I-III, capable of tolerating regional block procedures; (5) conscious, able to communicate without barriers, and able to comprehend and cooperate with pain assessment and respiratory function training.

Exclusion criteria were designed to control for confounding factors and ensure population homogeneity and safety, specifically including: (1) Known allergy or history of severe adverse reaction to amide-type local anesthetics (e.g., ropivacaine); (2) presence of skin breakdown, infection, severe deformity, or tumor at the intended puncture site; (3) documented coagulation dysfunction defined as International Normalized Ratio >1.5, or platelet count <80 × 10^9^/L, or current therapeutic anticoagulant/antiplatelet therapy that could not be safely paused; (4) concomitant severe trauma requiring urgent surgical intervention, such as severe traumatic brain injury, spinal cord injury with neurological deficit, or intra-abdominal organ rupture; (5) presence of bilateral rib fractures, flail chest, or immediate need for invasive mechanical ventilation due to respiratory failure; (6) history of chronic pain with regular long-term (>3 months) use of opioid analgesics or gabapentin/pregabalin; (7) pregnancy or lactation; (8) any other condition deemed by the investigator as unsuitable for participation based on clinical judgment, such as severe psychiatric disorder or expected survival <72 h.

### 2.3 Sample size and randomization

This study adopted an exploratory large-sample design. The primary objective was to provide precise effect estimates for a refined comparison of the two techniques and to ensure adequate statistical power for pre-specified subgroup analyses. The sample size was not fixed *a priori* but was based on the historical admission capacity of our Trauma Center, aiming to enroll as many eligible patients as possible within the predefined study period (December 1, 2023, to December 31, 2025). Ultimately, 158 patients completed randomization within this period (73 in the ESPB group, 85 in the SAPB group).

The randomization process was performed by a biostatistician independent of the research team. A block randomization sequence with a block length of 6 was generated using statistical software, allocating patients in a 1:1 ratio to the ESPB or SAPB group. This sequence was concealed in sequentially numbered, opaque, sealed envelopes kept by research nurses in each ward. The 1:1 block randomization sequence (block size of 6) was generated and applied correctly. However, the study terminated upon reaching the predefined end-of-study date (December 31, 2025) rather than upon completing a target sample size. Consequently, the final randomization block was partially enrolled at the time of study termination, resulting in an unequal group distribution (12 more patients allocated to SAPB than to ESPB). This type of imbalance is a known feature of block randomization when a trial ends before all blocks are fully filled. Importantly, the randomization sequence itself was not violated, and the intention-to-treat analysis remains valid.

### 2.4 Blinding

Successful double-blinding was implemented for patients and outcome assessors. The anesthesiologists performing the ultrasound-guided nerve blocks were aware of the group assignment to accurately execute the different technical protocols but did not participate in any subsequent data collection, assessment, or entry. To maintain patient blinding, all patients were informed they would receive “a standard ultrasound-guided analgesic treatment” and were placed in a separate procedure room. Following the procedure, the puncture site was covered with a uniform opaque dressing, preventing patient identification of the specific block type. All subsequent pain score assessments, respiratory function measurements, questionnaire administration, and clinical outcome documentation were conducted by dedicated research nurses or respiratory therapists completely blinded to group allocation. Data entry and analysis were also performed by statisticians unaware of group assignment.

### 2.5 Interventions

Standardized Baseline Analgesia: To eliminate interference from background analgesia, all enrolled patients received a uniform multimodal baseline analgesic regimen starting at admission: intravenous parecoxib sodium 8 mg (every 12 h) and acetaminophen 1 g (every 6 h). This regimen was continued before and after the intervention.

Ultrasound-Guided ESPB: Patients in the ESPB group were positioned in lateral decubitus with the non-affected side down or seated. A high-frequency linear ultrasound probe (frequency set to 8–13 MHz) was used. The operator performed a sagittal scan at the thoracic transverse process level corresponding to the area of most dense fractures (typically T4 to T7). The “mountain-peak” acoustic image of the transverse process tip and the overlying erector spinae muscle were clearly identified. Under strict aseptic conditions, using an in-plane technique with a 22-gauge, 80-mm needle inserted from the caudal side of the probe, the needle tip was advanced to the interface between the periosteum of the transverse process tip and the deep fascia of the erector spinae muscle. After negative aspiration confirmed absence of blood or air, 0.375% ropivacaine hydrochloride injection was slowly administered, with a total volume of 26.0 ± 3.5 mL. Under real-time ultrasound monitoring, the injectate was observed forming a characteristic spindle-shaped or linear hypoechoic shadow spreading cranially and caudally along the fascial plane between the transverse process and the muscle.

Ultrasound-Guided SAPB: Patients in the SAPB group were positioned in lateral decubitus with the affected side up, and the affected upper limb was abducted and elevated to fully expose the mid-axillary line. Using the same high-frequency linear probe placed at the level of the 4th or 5th intercostal space in the mid-axillary line, the latissimus dorsi, serratus anterior, intercostal muscles, and the underlying pleural line were identified from superficial to deep. Using an in-plane technique from the anterior aspect of the probe, the needle tip penetrated the serratus anterior fascia to reach the potential space between the layer between the latissimus dorsi and the serratus anterior. Similarly, after safety confirmation via negative aspiration, 0.375% ropivacaine hydrochloride was injected, with a total volume of 22.7 ± 3.0 mL. Ultrasound visualization showed the injectate separating the serratus anterior from the intercostal muscles and spreading within that plane.

All block procedures were performed by one attending anesthesiologist and one emergency room physician with anesthesia qualifications, each with over 5 years of experience in ultrasound-guided regional anesthesia and having independently performed at least 200 related procedures, to ensure technical consistency and success. Procedure time (from skin disinfection to needle withdrawal) was precisely recorded. *Rescue Analgesia Protocol: If a patient reported a resting pain NRS score >4 after the intervention, intravenous tramadol hydrochloride 50 mg was permitted as standardized rescue analgesia, with the time and dose of each administration meticulously recorded.

### 2.6 Observation indicators

*Primary outcome*: The primary outcome was a comprehensive measure of resting pain intensity over the 24 h post-intervention, specifically defined as the time-weighted area under the curve (AUC) of the resting NRS pain score from pre-intervention (T0) to 24 h post-intervention (T24). Pain was assessed at six pre-specified time points: T0 (immediately before block), T2, T4, T6, T12, and T24 h. The AUC was calculated using the trapezoidal rule. This metric comprehensively reflects both the intensity and durability of analgesic effect, providing more information than scores at single time points.

Secondary outcomes were categorized into several dimensions:

a) *Pain dimension*: Recording resting and cough-evoked NRS scores (assessed immediately after the patient performed a standard deep-inspiration cough) at the aforementioned time points.
b) *Respiratory function dimension*: At T0 and T24, using bedside ultrasound to measure the excursion amplitude of the ipsilateral hemidiaphragm during quiet and deep breathing; using a calibrated portable spirometer to measure Forced Vital Capacity (FVC) and Forced Expiratory Volume in the first second (FEV1). All spirometry measurements were performed by trained respiratory therapists under strict safety monitoring. The following criteria were applied: Spirometry was only performed if the patient’s resting pain NRS score was ≤6 after the block procedure; patients were instructed to perform forced expiratory maneuvers gradually, starting with a gentle exhalation followed by a maximal forced effort, and were explicitly allowed to stop at any time if they experienced intolerable pain or discomfort; a research nurse monitored patients for any signs of clinical deterioration during the maneuver, including sudden increase in pain, respiratory distress, oxygen desaturation (SpO₂ < 90%), or hemodynamic instability; spirometry was immediately terminated if the patient requested to stop or if any of the above adverse signs occurred. Importantly, no patient in either group experienced procedure-related complications (e.g., pneumothorax aggravation, new-onset hemoptysis, or hemodynamic compromise) during or immediately after the forced expiratory maneuvers. Additionally, no test was interrupted or deemed unperformable due to uncontrollable pain, as all patients had achieved adequate analgesia (resting NRS ≤ 4 post-intervention) prior to the T24 assessment.
c) *Pharmacoeconomic and recovery quality dimension*: Precisely calculating the total consumption of rescue opioid (tramadol) within 24 hours post-intervention, standardized by conversion to oral morphine milligram equivalents (MME); quantitatively assessing overall postoperative recovery comfort and functional status at T24 using the validated Chinese version of the 15-item Quality of Recovery scale (QoR-15).
d) *Clinical hard endpoint dimension*: Recording the occurrence of pulmonary complications (e.g., pneumonia, radiologically confirmed atelectasis) during hospitalization, total hospital length of stay, and whether intensive care unit (ICU) admission occurred and its duration.
e) *Safety dimension*: Closely monitoring and recording any adverse events directly or potentially related to the block procedure, including but not limited to local hematoma, infection, systemic local anesthetic toxicity from intravascular injection, and pneumothorax.
f) *Pre-specified subgroup analysis*: To explore heterogeneity of treatment effect, an analysis was pre-specified based on anatomical rationale, stratifying by the key factor of dominant fracture location, to compare the analgesic effect difference between ESPB and SAPB in patients categorized as having “predominantly anterolateral fractures” versus “predominantly posterior fractures.”

### 2.7 Statistical analysis

All statistical analyses were performed using SPSS version 27.0 (Chinese version) and followed the intention-to-treat principle, whereby all randomized patients were analyzed in their originally assigned groups.

For continuous variables, normality was first assessed using the Shapiro–Wilk test. Data conforming to a normal distribution are presented as mean ± standard deviation and compared between groups using independent samples t-tests. Non-normally distributed data are presented as median and interquartile range and compared using the Mann–Whitney U test. For repeatedly measured continuous variables, repeated-measures analysis of variance was used. Categorical variables are presented as frequency and percentage and compared between groups using chi-square or Fisher’s exact tests.

For the primary outcome (pain AUC), a single pre-specified endpoint was tested, so no adjustment for multiple comparisons was required. For the secondary outcomes, which included repeated measurements over multiple time points and multiple domains (pain, respiratory function, opioid consumption, quality of recovery, and clinical outcomes), the analyses were primarily exploratory and hypothesis-generating in nature. Therefore, no formal correction for multiple comparisons (e.g., Bonferroni adjustment) was applied to control the family-wise type I error rate across secondary analyses. Instead, the interpretation of secondary outcomes is based on effect sizes and their 95% confidence intervals, with *p*-values provided as descriptive measures of evidence. This approach is consistent with the CONSORT 2010 statement recommendation for reporting secondary outcomes in confirmatory trials, which emphasizes that results for secondary outcomes should be interpreted cautiously and are best viewed as supportive evidence for the primary findings.

## 3 Results

### 3.1 Participant enrollment process and comparison of baseline characteristics

**Figure 1:**
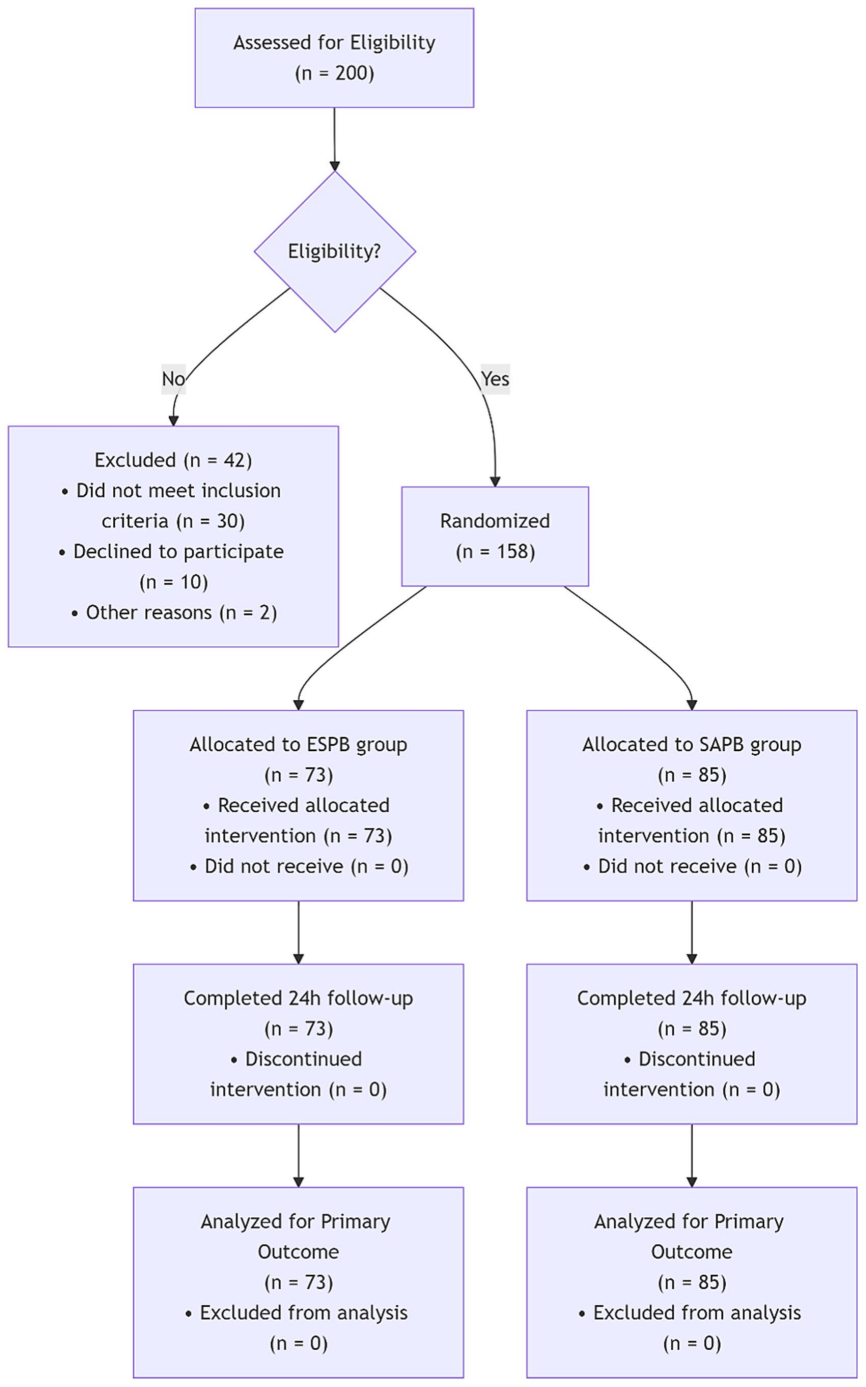
Participant flow diagram (CONSORT). A total of 200 patients were screened, of whom 158 met the inclusion criteria and were randomized to receive either erector spinae plane block (ESPB, *n* = 73) or serratus anterior plane block (SAPB, *n* = 85). All randomized patients received the allocated intervention, completed the 24 h follow-up without loss or discontinuation, and were included in the intention-to-treat analysis. No patients were excluded from the primary analysis.

The study strictly adhered to the pre-defined protocol. A total of 200 patients were screened. Among them, 158 patients with unilateral multiple rib fractures who met the inclusion and exclusion criteria were successfully randomized and all were included in the final intention-to-treat analysis, with no dropouts. The detailed flow of participants is shown in the CONSORT diagram (Figure 1). A comprehensive comparison of baseline characteristics between the two groups (Table 1) revealed no statistically significant differences in demographic features, including age, sex distribution, and body mass index (all *p* > 0.05). The composition ratios of American Society of Anesthesiologists physical status classification were also similar, indicating that the overall physiological status and anesthesia risk of the two groups were essentially equivalent. Regarding key indicators of trauma severity, the two groups were highly matched in the average number of rib fractures, the distribution proportion of the dominant fracture location (anterolateral or posterior), and the incidence of concomitant pulmonary contusion (all *p* > 0.05). Crucially, no significant differences were observed between the groups in the baseline functional status assessed immediately pre-intervention, including resting and cough pain intensity, diaphragmatic excursion, forced vital capacity (FVC), and forced expiratory volume in the first second (FEV1) (all *p* > 0.05). This established a solid foundation of comparability for subsequent between-group efficacy comparisons. The sole operational difference was that, according to the technical specifications, the volume of local anesthetic injected was significantly greater in the ESPB group than in the SAPB group (25.94 ± 3.94 mL vs. 22.61 ± 4.39 mL, *p* < 0.001), reflecting the inherent anatomical and pharmacological characteristics of the two techniques. There was no difference in the block procedure time between the two groups (16.84 ± 5.70 min vs. 17.75 ± 6.12 min, *p* = 0.338).

**Table 1:** Comparison of baseline characteristics and procedural details between groups.

Characteristic	ESPB group (*n* = 73)	SAPB group (*n* = 85)	*p*-value
Demographics			
Age (years), mean ± SD	50.48 ± 14.97	52.29 ± 14.14	0.435
Male, *n* (%)	43 (58.9)	59 (69.4)	0.169
Body mass index (kg/m^2^), mean ± SD	24.72 ± 3.62	24.64 ± 3.66	0.889
ASA physical status (I/II/III), *n*	20/29/24	26/28/31	0.676
Injury characteristics			
Total rib fractures, mean ± SD	5.16 ± 1.55	4.84 ± 1.48	0.174
Dominant fracture location, *n* (%)			0.397
Predominantly anterolateral	32 (43.8)	43 (50.6)	
Predominantly posterior	41 (56.2)	42 (49.4)	
Pulmonary contusion, *n* (%)	29 (39.7)	36 (42.4)	0.738
Procedural details			
Local anesthetic volume (mL), mean ± SD	25.94 ± 3.94	22.61 ± 4.39	**<0.001**
Baseline functional assessment			
Resting pain NRS (0–10), mean ± SD	7.48 ± 1.35	7.44 ± 1.38	0.871
Cough pain NRS (0–10), mean ± SD	8.71 ± 1.02	8.47 ± 1.18	0.172
Diaphragmatic excursion (mm), mean ± SD	35.26 ± 6.31	37.15 ± 6.47	0.065
Forced vital capacity, FVC (L), mean ± SD	2.57 ± 0.68	2.51 ± 0.55	0.542
FEV1 (L), mean ± SD	2.04 ± 0.51	2.02 ± 0.45	0.782

ESPB, erector spinae plane block; SAPB, serratus anterior plane block; ASA, American society of anesthesiologists; NRS, Numerical Rating Scale; FEV1, forced expiratory volume in the first second. Data presented as mean ± standard deviation or *n* (%). *p*-values from independent *t*-test or *χ^2^*/Fisher’s exact test. Bold values indicate statistically significant differences (*p* < 0.05).

### 3.2 Primary efficacy endpoint: 24-hour comprehensive analgesic effect

**Figure 2:**
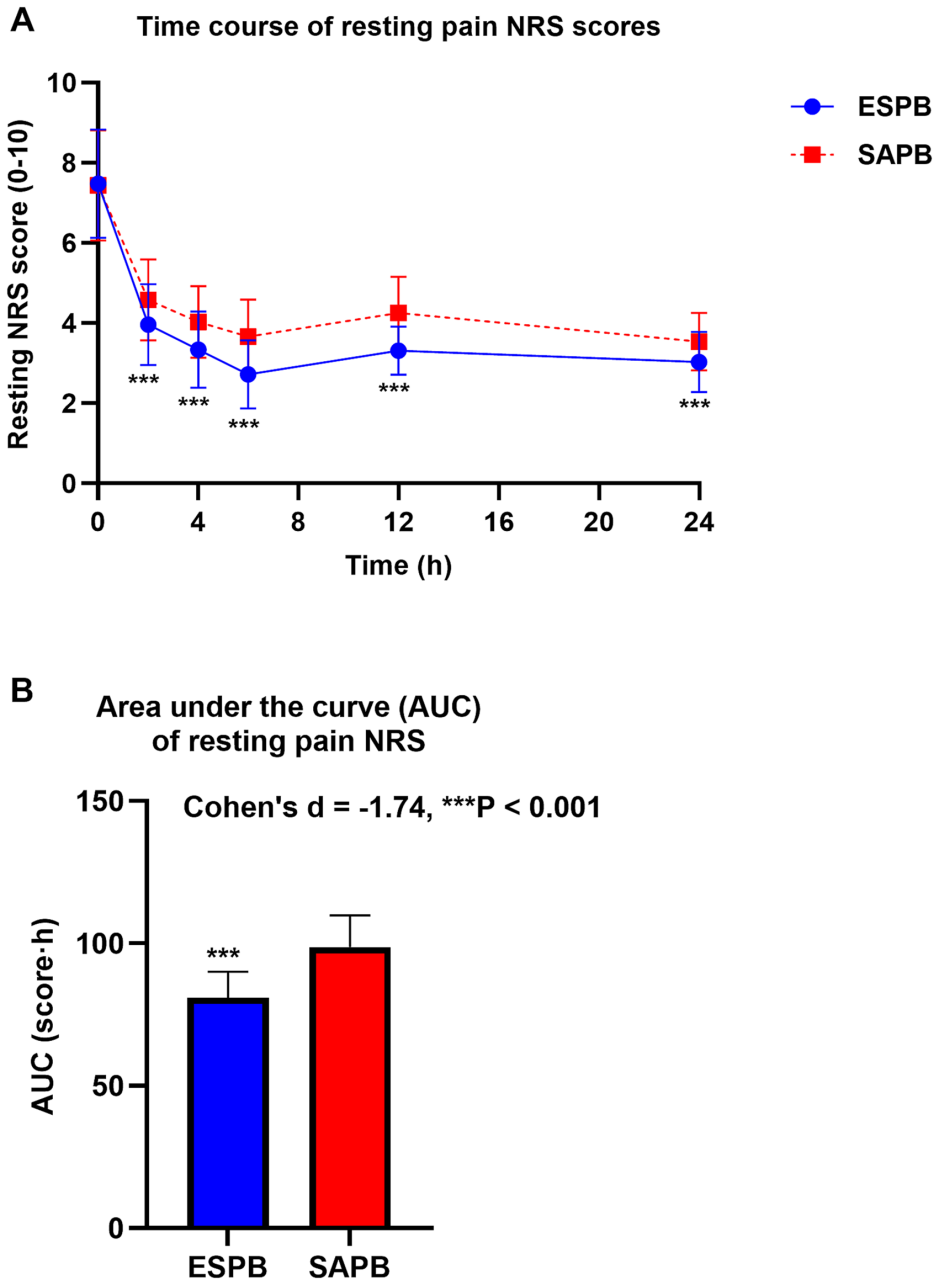
Comparison of 24-h analgesic efficacy between ESPB and SAPB groups. **(A)** Time course of resting pain NRS scores at 0, 2, 4, 6, 12, and 24 h after nerve block. **(B)** Area under the curve (AUC) of resting pain NRS scores over 24 h. Data are presented as mean ± SD. ***p < 0.001 vs. SAPB group by independent *t*-test. ESPB: Erector spinae plane block; SAPB: Serratus anterior plane block.

Analysis of the primary outcome measure—the area under the curve (AUC) of resting pain scores over 24 h—showed homogeneity of variance between the two datasets (*p* = 0.084). The pain burden was significantly lower in the ESPB group compared to the SAPB group. Specifically, the mean AUC was 80.96 (SD 9.13) in the ESPB group versus 98.74 (SD 11.07) in the SAPB group. Independent samples t-test revealed a mean difference of −17.78 (95% CI: −21.00 to −14.56) between the groups, a difference that was highly statistically significant (*t* = −10.903, *p* < 0.001). Effect size analysis further indicated a very large effect size for the difference between the groups (Cohen’s d = −1.74, 95% CI: −2.11 to −1.37). This result confirms, from a holistic temporal dimension, that the erector spinae plane block provides sustained analgesic efficacy far superior to the serratus anterior plane block for patients with multiple rib fractures. As shown in Figure 2, starting from 2 h post-block, the resting pain scores in the ESPB group were consistently lower than those in the SAPB group, and this advantage was maintained throughout the 24-h observation period.

### 3.3 Analysis of secondary efficacy endpoints

#### 3.3.1 Dynamic pain relief and opioid-sparing effect

**Figure 3:**
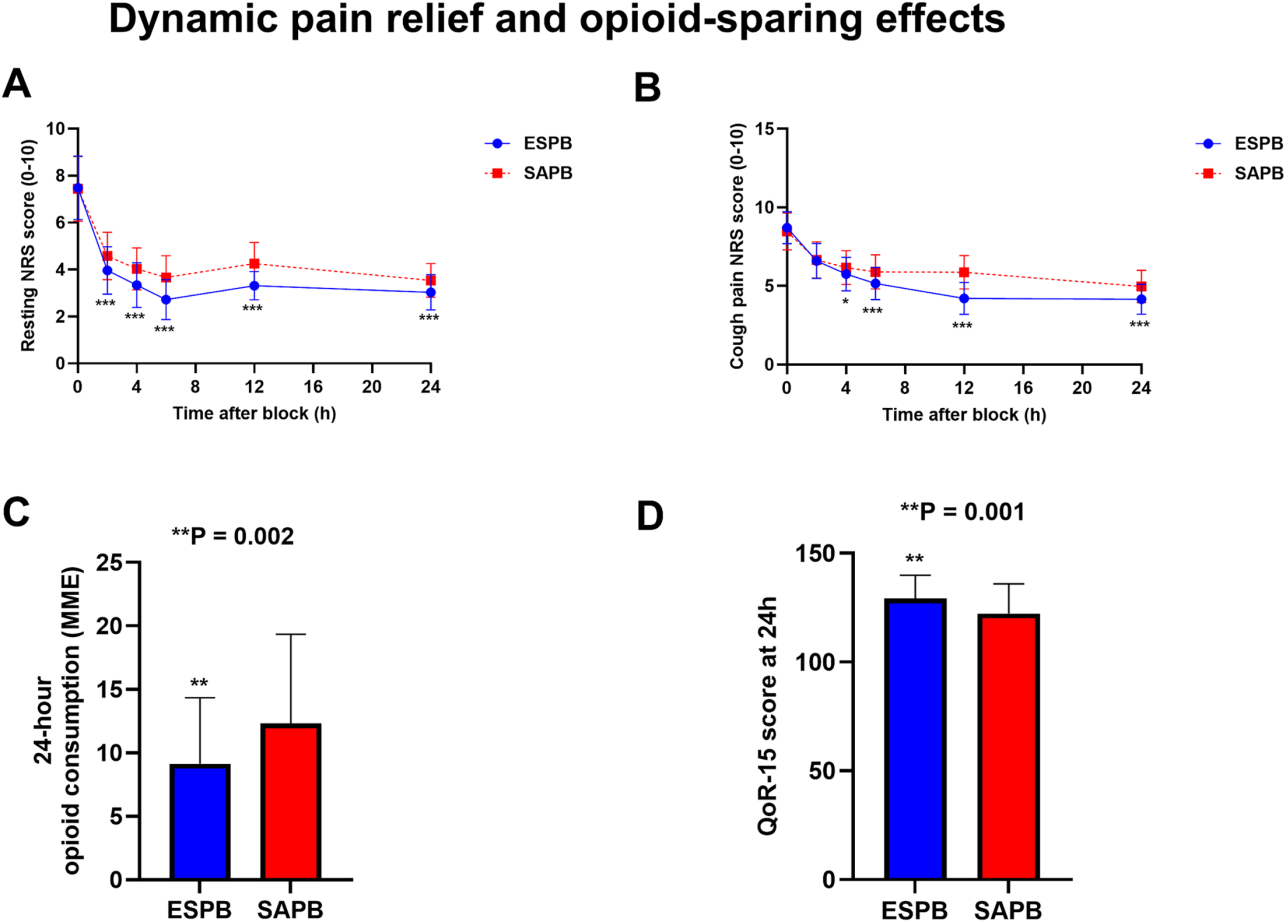
Dynamic pain relief and opioid-sparing effects of ESPB compared to SAPB in patients with multiple rib fractures. **(A)** Time course of resting pain NRS scores. **(B)** Time course of cough pain NRS scores. **(C)** 24-h opioid consumption expressed as morphine milligram equivalents (MME). **(D)** Quality of Recovery-15 (QoR-15) scores at 24 h after nerve block. Data are presented as mean ± SD. *p < 0.05, **p < 0.01, ***p < 0.001 vs. SAPB group. ESPB: Erector spinae plane block; SAPB: Serratus anterior plane block.

Beyond the primary endpoint, dynamic pain assessment at each pre-specified time point further delineated the analgesic profiles of the two techniques. As shown in Figure 3, regarding resting pain, the ESPB group demonstrated significantly lower pain scores at all assessment points postoperatively: 2, 4, 6, 12, and 24 h (all *p* < 0.001). For cough pain, which better reflects functional status, the advantage of the ESPB group became apparent from 4 h postoperatively and persisted at 6, 12, and 24 h (*p* < 0.05). Echoing the superior analgesic effect was a significant opioid-sparing effect. The mean consumption of rescue opioids (converted to morphine milligram equivalents) within 24 h postoperatively was 9.13 mg in the ESPB group, significantly lower than the 12.33 mg in the SAPB group (*p* = 0.002). Furthermore, assessment using the Quality of Recovery-15 scale showed that the overall recovery quality score at 24 h postoperatively was also significantly higher in the ESPB group than in the SAPB group (129.23 vs. 122.32, *p* = 0.001), suggesting that better pain control is directly associated with improved subjective patient recovery experience (Table 2).

**Table 2:** Comparison of opioid consumption, recovery quality, and key respiratory outcomes at 24 hours post-intervention.

Outcome measure	ESPB group (*n* = 73)	SAPB group (*n* = 85)	*p*-value
24-h opioid consumption (MME), mean ± SD	9.13 ± 5.21	12.33 ± 7.01	**0.002**
QoR-15 score at T24, mean ± SD	129.23 ± 10.65	122.32 ± 13.56	**0.001**
Diaphragmatic excursion at T24 (mm), mean ± SD	47.94 ± 7.40	48.26 ± 6.31	0.771
FVC at T24 (L), mean ± SD	3.19 ± 0.59	3.07 ± 0.53	0.175

ESPB, erector spinae plane block; SAPB, serratus anterior plane block; MME, oral morphine milligram equivalents; QoR-15, 15-item quality of recovery scale; FVC, forced vital capacity; T24, 24 h post-intervention. *p*-values from independent *t*-test. Bold values indicate statistically significant differences (*p* < 0.05).

#### 3.3.2 Improvement in objective respiratory function indicators

**Figure 4:**
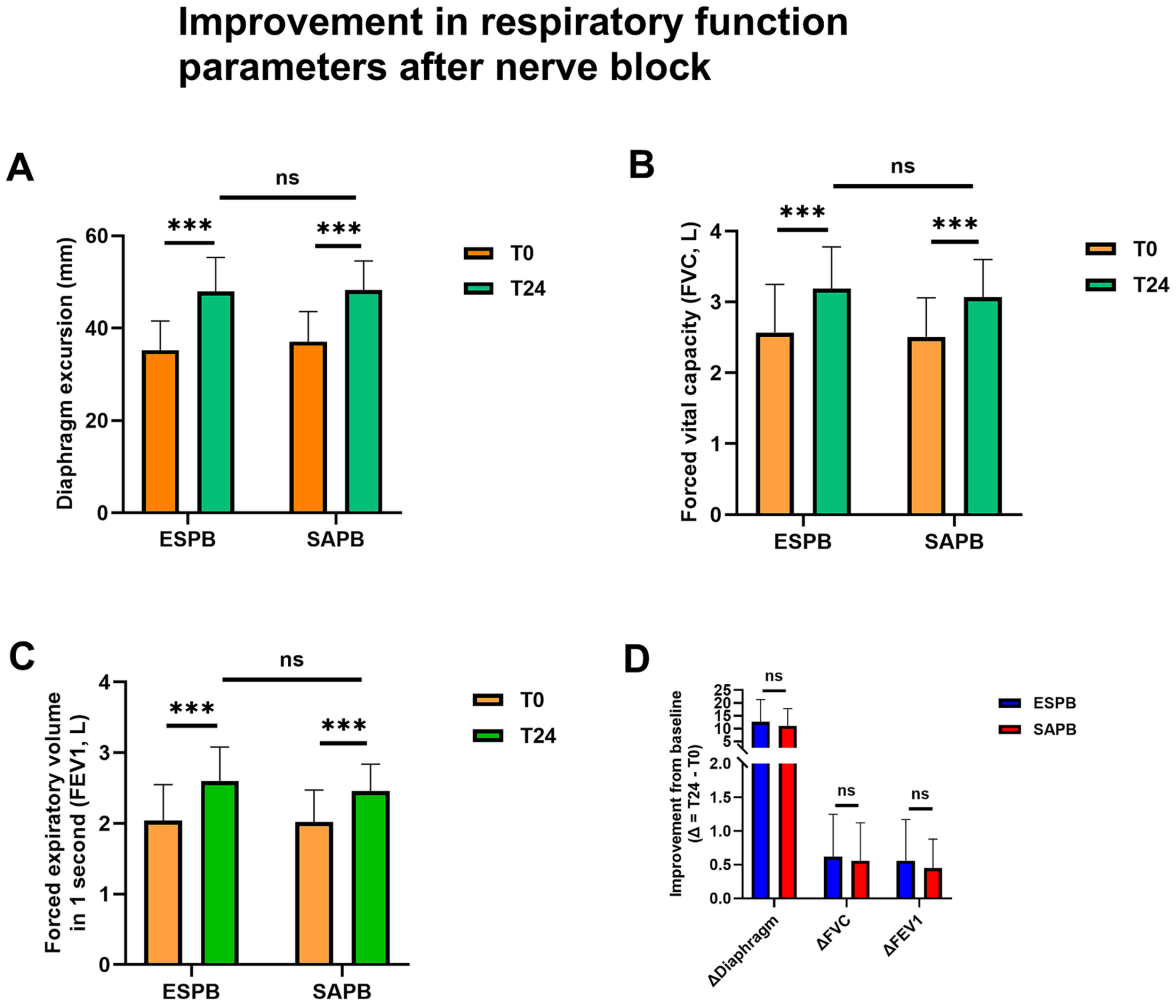
Comparison of respiratory function improvement between ESPB and SAPB groups. **(A)** Diaphragm excursion measured by ultrasound. **(B)** Forced vital capacity (FVC). **(C)** Forced expiratory volume in 1 s (FEV1). **(D)** Improvement from baseline (*Δ* = T24—T0). Data are presented as mean ± SD. ***p < 0.001 for within-group comparison (T0 vs. T24); ns: not significant for between-group comparison at T24. ESPB: Erector spinae plane block; SAPB: Serratus anterior plane block.

Effective pain management forms the basis for restoring respiratory function. Objective data measured by ultrasound and spirometer in this study showed that the respiratory function of patients in both groups improved significantly from baseline by 24 h post-intervention. As shown in Figure 4, diaphragmatic excursion, FVC, and FEV1 showed significant improvement from baseline in both groups (within-group comparison, all *p* < 0.001). However, between-group comparison at the T24 time point revealed no statistically significant differences in the degree of improvement for diaphragmatic excursion (47.94 ± 7.40 mm vs. 48.26 ± 6.31 mm, *p* = 0.771) or FVC (3.19 ± 0.59 L vs. 3.07 ± 0.53 L, *p* = 0.175). The difference in FEV1 also did not reach statistical significance (2.60 ± 0.48 L vs. 2.46 ± 0.38 L, *p* = 0.054). Analysis of the improvement values showed that although the absolute improvement values for diaphragmatic excursion (12.68 ± 8.65 mm vs. 11.11 ± 6.69 mm), FVC (0.62 ± 0.63 L vs. 0.56 ± 0.56 L), and FEV1 (0.56 ± 0.61 L vs. 0.45 ± 0.43 L) were slightly higher in the ESPB group than in the SAPB group, independent samples *t*-tests indicated that none of these differences reached statistical significance (*p*-values were 0.199, 0.518, and 0.193, respectively). The 95% CIs for the improvement values all included zero, further supporting the conclusion that the degree of respiratory function improvement was comparable between the groups. Of note, all 158 patients tolerated the spirometry maneuvers without requiring premature termination. No adverse events (e.g., increased chest pain, oxygen desaturation, hemoptysis, or suspected pneumothorax exacerbation) were observed during or immediately after the forced expiratory maneuvers.

**Figure 5:**
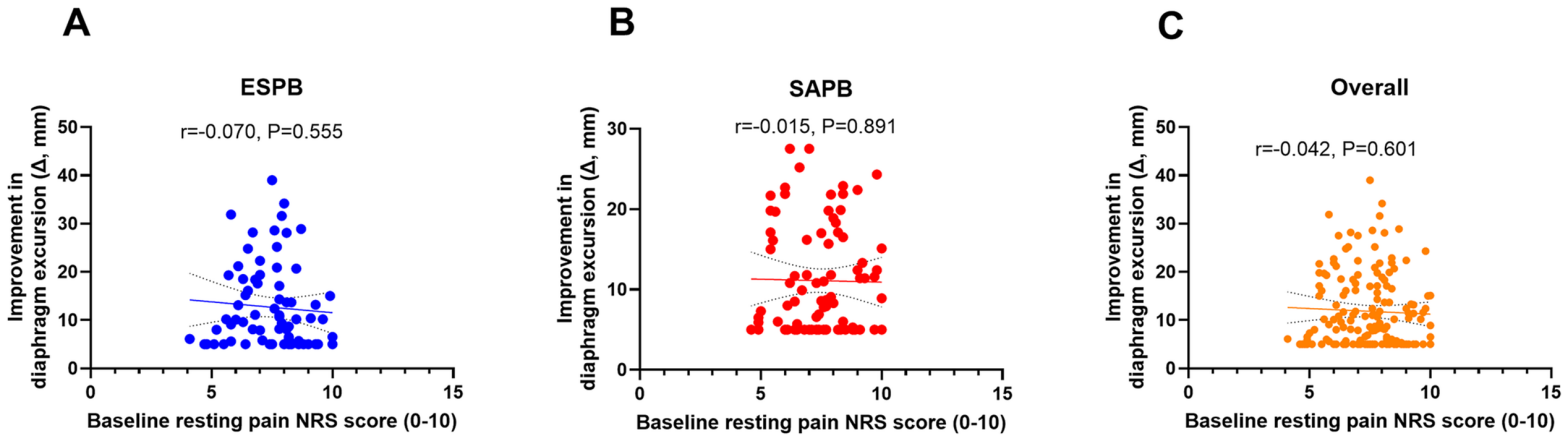
Scatter plots demonstrating the lack of significant correlation between baseline resting pain NRS scores and improvement in diaphragm excursion following nerve block. **(A, B)** Group-specific correlations for ESPB (blue circles, *r* = −0.070, *p* = 0.555) and SAPB (red squares, *r* = −0.015, *p* = 0.891). Solid lines represent linear regression fits with 95% confidence bands (shaded areas). **(C)** Overall correlation including all 158 patients (*r* = −0.042, *p* = 0.601). The near-zero correlation coefficients (all |*r*| < 0.1) and non-significant *p* values (all > 0.05) indicate that baseline pain intensity does not predict the degree of diaphragmatic improvement in this patient population.

Correlation analysis revealed no significant linear relationship between the baseline resting pain NRS score and the improvement in diaphragmatic excursion (*Δ* = T24—T0). The overall correlation coefficient was *r* = −0.042 (*p* = 0.601), with a coefficient of determination *R^2^* = 0.0018, indicating that baseline pain level explained only 2% of the variance in diaphragmatic function improvement. Stratified analysis by block type showed no significant correlation observed in either the ESPB group (*r* = −0.07, *p* = 0.555) or the SAPB group (*r* = −0.015, *p* = 0.891) (Figure 5). This may suggest that once pain is effectively alleviated beyond a certain threshold, further intensification of analgesia may have a plateau effect on the direct promotion of respiratory muscle function.

#### 3.3.3 Analysis of treatment effect heterogeneity based on fracture characteristics

**Figure 6:**
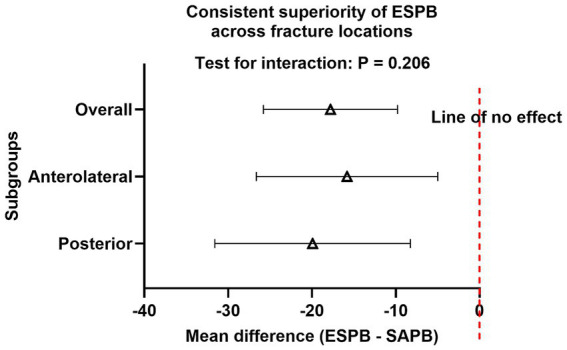
Forest plot of subgroup analysis by fracture location showing consistent superiority of ESPB over SAPB. Diamonds represent mean differences in pain AUC with 95% confidence intervals. The vertical dashed line at zero indicates no difference between treatments. Posterior fractures: MD = −19.92 (−24.62 to −15.23), *p* < 0.001; Anterolateral fractures: MD = −15.81 (−20.16 to −11.45), *p* < 0.001. Test for interaction: *p* = 0.206. ESPB, Erector spinae plane block; SAPB, Serratus anterior plane block; AUC, Area under the curve; MD, Mean difference.

**Figure 7:**
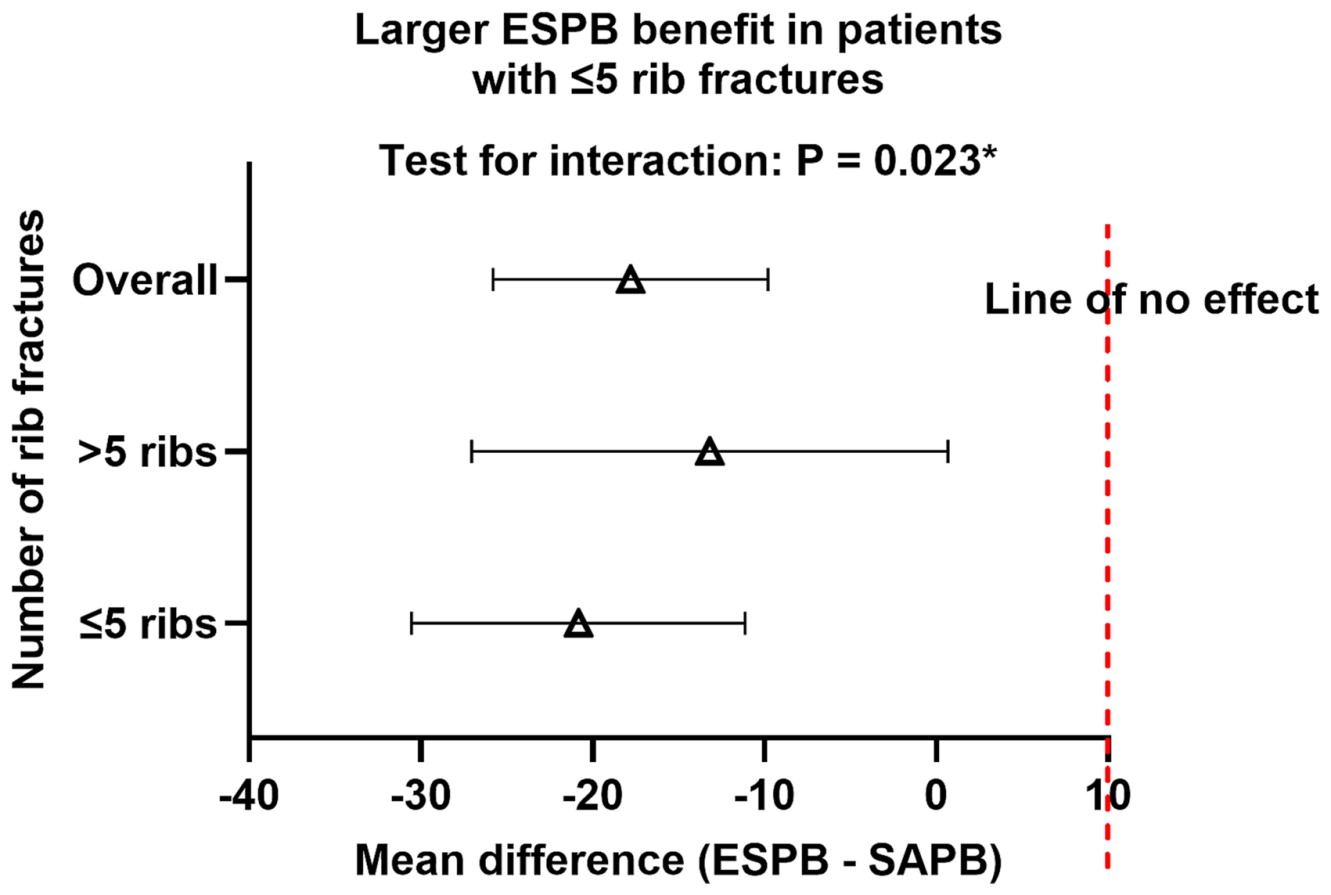
Forest plot of subgroup analysis by number of rib fractures showing significant interaction (*p* = 0.023). The vertical dashed line at zero indicates no difference between treatments. ≤5 fractures: MD = −20.83 (−24.74 to −16.92), *p* < 0.001, Cohen’s d = −2.16; >5 fractures: MD = −13.19 (−18.77 to −7.61), *p* < 0.001, Cohen’s *d* = −1.22. Interaction effect: 7.64 (1.08 to 14.21), *p* = 0.023. ESPB, Erector spinae plane block; SAPB, Serratus anterior plane block; MD, Mean difference.

The pre-specified subgroup analysis provided crucial insights for individualized treatment selection. Two-way analysis of variance revealed a significant main effect of treatment group on resting pain AUC (*p* < 0.001), and a marginal effect of dominant fracture location on pain (*p* = 0.076). Critically, the interaction effect between treatment group and dominant fracture location did not reach statistical significance (*p* = 0.206). Specific results of the subgroup analysis showed that in patients with predominantly posterior fractures, the resting pain AUC was significantly lower in the ESPB group (81.32 ± 10.57) than in the SAPB group (101.25 ± 10.91), with a mean difference of −19.92 (95% CI: −24.62 to −15.23, *p* < 0.001). In patients with predominantly anterolateral fractures, the resting pain AUC was also significantly lower in the ESPB group (80.49 ± 7.01) than in the SAPB group (96.29 ± 10.78), with a mean difference of −15.81 (95% CI: −20.16 to −11.45, *p* < 0.001). Although the numerical advantage of ESPB was greater in the posterior fracture subgroup than in the anterolateral subgroup (interaction effect = −4.115, 95% CI: −10.52 to 2.29), this difference was not statistically significant (*p* = 0.206), suggesting that the analgesic superiority of ESPB was consistent across patients with different fracture locations (Figure 6). Furthermore, for patients with ≤5 fractures (*n* = 98), the resting pain AUC was significantly lower in the ESPB group (78.71 ± 7.96) than in the SAPB group (99.54 ± 10.74), with a mean difference of −20.83 (95% CI: −24.74 to −16.92, *p* < 0.001). For patients with >5 fractures (*n* = 60), the ESPB group (84.00 ± 9.85) was also significantly superior to the SAPB group (97.19 ± 11.70), but the magnitude of advantage was smaller, with a mean difference of −13.19 (95% CI: −18.77 to −7.61, *p* < 0.001). The interaction effect value was 7.64 (95% CI: 1.08 to 14.21, *p* = 0.023), indicating that patients with fewer fractures derived greater additional analgesic benefit from ESPB (Figure 7).

#### 3.3.4 Inpatient clinical outcomes and safety evaluation

**Figure 8:**
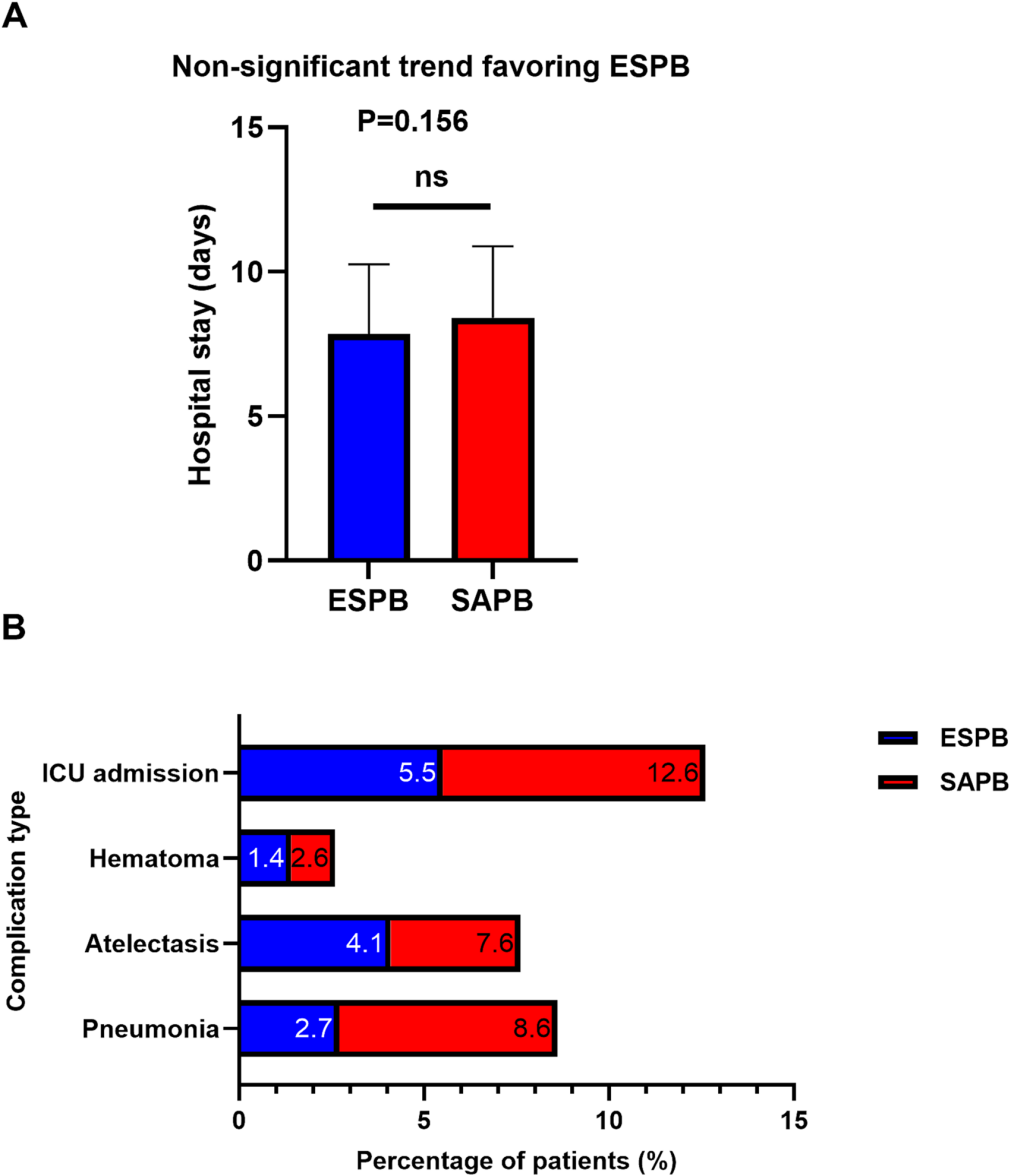
Comparison of hospital clinical outcomes and safety between erector spinae plane block (ESPB) and serratus anterior plane block (SAPB) groups. **(A)** Length of hospital stay, showing a non-significant trend toward shorter hospitalization in the ESPB group (mean difference: −0.56 days, *p* = 0.156). **(B)** Incidence of specific complications, demonstrating low and comparable rates in both groups. All statistical comparisons showed no significant differences (all *p* > 0.05), supporting the equivalent safety profile of ESPB compared to SAPB.

Regarding final clinical hard endpoints, this study observed a positive trend. The mean hospital length of stay was approximately 0.56 days shorter in the ESPB group compared to the SAPB group, although this difference did not reach statistical significance (*p* = 0.156). In terms of complications (Figure 8, Table 3), the proportions of patients developing pulmonary complications such as pneumonia and atelectasis were low in both groups, with no significant between-group differences. The incidence of block-related adverse events (e.g., puncture site hematoma) was very low and comparable between groups, and no serious adverse events such as systemic local anesthetic toxicity occurred. These data collectively indicate that while providing superior analgesia, ESPB did not introduce additional clinical risks, and its safety profile was comparable to that of SAPB.

**Table 3:** Comparison of hospital stay and adverse events between groups.

Outcome	ESPB group (*n* = 73)	SAPB group (*n* = 85)	*p*-value
Hospital length of stay (days), mean ± SD	7.84 ± 2.41	8.40 ± 2.48	0.156
Complications, *n* (%)			
Pneumonia	2 (2.7)	5 (5.9)	0.339
Atelectasis	3 (4.1)	3 (3.5)	0.849
Adverse events, *n* (%)			
Puncture site hematoma	1 (1.4)	1 (1.2)	0.914
Other outcomes, *n* (%)			
ICU admission	4 (5.5)	6 (7.1)	0.684

ESPB, erector spinae plane block; SAPB, serratus anterior plane block; ICU, Intensive Care Unit. *p*-values from independent *t*-test, *χ^2^* or Fisher’s exact test.

## 4 Discussion

This study directly compared the efficacy of ultrasound-guided ESPB versus SAPB in patients with acute unilateral multiple rib fractures through a prospective, randomized, double-blind controlled trial with a large single-center sample. Our findings strongly support the primary hypothesis: compared to SAPB, ESPB provides significantly superior early comprehensive analgesia, effectively reduces opioid requirements, and enhances patient self-reported recovery quality. Particularly importantly, the subgroup analysis not only clarifies previous inferences but also identifies a new, statistically significant moderator of treatment effect—the number of fractures—providing a richer basis for refined clinical decision-making.

The significant advantage of ESPB in 24-h pain burden fundamentally stems from the distinct pharmacological diffusion mechanisms and anatomical targets of the two techniques (16). ESPB involves injecting local anesthetic into the fascial interface between the thoracic transverse processes and the erector spinae muscle. The solution can spread extensively along this plane and may permeate into the paravertebral space, thereby blocking both the dorsal and ventral rami (i.e., intercostal nerves) of the spinal nerves. This relatively “central” injection strategy enables it to cover a broad area extending from the posterior paraspinal region to the lateral chest wall. In contrast, SAPB, as a more “peripheral” plane block, primarily confines the solution to the serratus anterior plane, mainly affecting the lateral cutaneous branches of the intercostal nerves (17). It provides definitive analgesia for the anterolateral chest wall but offers limited coverage of the posterior region innervated by the dorsal rami. Although based on anatomy, we hypothesized that the analgesic superiority of ESPB would be more pronounced for posterior fractures, the subgroup analysis of this study showed that the interaction between treatment group and dominant fracture location did not reach statistical significance. This indicates that the analgesic advantage of ESPB relative to SAPB is stably present and similar in degree in both patients with anterolateral and posterior fractures (18). This finding expands the understanding of the applicable population for ESPB, suggesting its broad neural coverage capability can deliver excellent analgesic effects across different anatomical fracture patterns.

A more enlightening discovery is that the number of fractures significantly moderates the difference in efficacy between the two block techniques (19). The interaction analysis indicates that for patients with fewer fractures (≤5), the additional analgesic benefit brought by ESPB is more prominent. A possible mechanism is that within a relatively confined traumatic area, the extensive and concentrated neural blockade provided by ESPB can more completely cover the primary pain regions. In contrast, for more extensive and diffuse multiple fractures (>5), a single plane block might struggle to completely block all involved neural segments, leading to a somewhat reduced absolute analgesic gap between the two techniques. This finding suggests that in clinical decision-making, besides considering fracture location, the extent (number) of fractures should also serve as an important dimension for consideration.

Although ESPB demonstrated superior analgesic potency, both techniques ultimately achieved comparable improvements in objective respiratory function indicators, including diaphragmatic excursion and lung volumes. Furthermore, baseline pain level showed no linear correlation with the magnitude of diaphragmatic function improvement. Collectively, these findings support the existence of an analgesic “effective threshold” for respiratory recovery following rib fractures (20). We propose that once pain is alleviated below a certain critical threshold by either regional block—sufficient to enable patients to perform effective deep breathing and coughing—the primary mechanical impediments to respiration are overcome. Beyond this threshold, further incremental differences in analgesic intensity may yield diminishing returns on further improvements in diaphragmatic excursion or lung volumes (21). In this study, both the ESPB and SAPB groups achieved this threshold, as evidenced by the significant within-group improvements in respiratory parameters from baseline (all *p* < 0.001). Consequently, the superior analgesic efficacy of ESPB (lower pain AUC and reduced opioid consumption) did not translate into statistically greater respiratory function gains; instead, these additional benefits were manifested in other clinically meaningful domains, including enhanced patient comfort, reduced opioid-related side effects, and superior overall recovery quality, as reflected by significantly higher QoR-15 scores (22).

Regarding clinical outcomes, the ESPB group showed a trend towards shorter hospital length of stay. Although not statistically significant, this positive signal warrants attention. Better pain control may facilitate earlier patient mobilization and cooperation with rehabilitation (23). Both groups in this study exhibited low and comparable rates of adverse events, reaffirming the high procedural safety of these two ultrasound-guided fascial plane block techniques (24).

Certainly, this study has several limitations. The single-center design ensures internal consistency but may affect the generalizability of the conclusions (25). Complete blinding of operators was not possible, although standardized procedures aimed to control for this. Additionally, although a 1:1 block randomization was employed, the final group allocation was not perfectly balanced (73 vs. 85) due to study termination at a predefined end-of-study date before completing the final randomization block. While this imbalance is unlikely to bias the intention-to-treat estimates, it represents a minor deviation from perfect allocation equality. The study focused on acute-phase outcomes and did not assess long-term impact on chronic pain. Furthermore, local anesthetic volume could not be standardized due to the technical principles of each block (26), but this accurately reflects clinical practice. While the differences in opioid consumption (3.2 MME) and pain AUC (a mean difference corresponding to ~0.74 points on the 0–10 NRS scale) were statistically significant, their magnitudes may approach the lower boundary of what is considered the minimal clinically important difference (MCID) for individual patients. However, we argue that the cumulative benefits—including a 26% relative reduction in rescue opioid requirements and significantly improved patient-reported quality of recovery (QoR-15)—collectively support the clinical preference for ESPB, particularly in patients with ≤5 rib fractures where the treatment effect was largest. Moreover, the volume of local anesthetic administered differed between the two groups (25.94 ± 3.94 mL for ESPB vs. 22.61 ± 4.39 mL for SAPB, *p* < 0.001), reflecting the distinct anatomical and pharmacokinetic requirements of each block technique. While this difference is clinically justified—ESPB typically requires larger volumes to achieve adequate craniocaudal fascial spread—it may nevertheless act as a potential confounding factor when attributing the superior analgesic efficacy solely to the anatomical target of ESPB. Future studies could explore whether volume-adjusted comparisons (e.g., using weight-based or equal-volume protocols) yield similar results. Future research could be conducted in a multicenter setting, incorporate longer-term follow-up, and further explore the value of combined blocks or catheter techniques in patients with the most extensive fractures.

## 5 Conclusion

In summary, for unilateral multiple rib fractures, ultrasound-guided ESPB demonstrates significantly superior overall analgesic efficacy compared to SAPB” (kept as factual statement), and this advantage is consistent across patients with different fracture locations. Therefore, when conditions permit, the ESPB may be prioritized as a first-line analgesic strategy. Particularly, for patients with a relatively lower number of fractures (≤5), choosing the ESPB may yield greater additional analgesic benefit. This clinical decision should comprehensively consider the patient’s specific fracture pattern, operator experience, and available clinical resources, aiming to maximize the benefits of pain management and provide the optimal solution for promoting early patient recovery and improving outcomes. Nevertheless, the SAPB retains clinical utility in specific circumstances. SAPB remains an appropriate first-line choice for patients with predominantly anterolateral fractures when operator experience favors this approach, in the presence of posterior chest wall trauma that precludes a safe ESPB, or when continuous catheter techniques are planned in settings where managing a posterior catheter is logistically challenging. However, when conditions permit, ESPB may be considered as a preferred first-line regional analgesic technique.

## Data Availability

The datasets presented in this study can be found in online repositories. The names of the repository/repositories and accession number(s) can be found in the article/supplementary material.
